# An Exploration of Vitamin D Deficiency and Clinical Status in Friedreich's Ataxia Patients in the UK

**DOI:** 10.1002/mdc3.70529

**Published:** 2026-03-06

**Authors:** Zofia Fleszar, Gilbert Thomas‐Black, Hector Garcia‐Moreno, Arron Cook, Paola Giunti

**Affiliations:** ^1^ Department of Clinical and Movement Neurosciences UCL Queen Square Institute of Neurology London UK

**Keywords:** Freidreich's ataxia, vitamin D

Vitamin D deficiency has been described in several neurodegenerative conditions. Friedreich's ataxia (FRDA) is the most common inherited neurodegenerative ataxia, characterized by progressive gait, limb and trunk ataxia, together with peripheral neuropathy, pes cavus, skeletal deformities, and cardiomyopathy. The result is a highly disabling disease in which early falls are common, and wheelchair use is common by the third decade.^1^ There is a lack of evidence looking at bone health in FRDA and so the aim of the current study was to review the vitamin D status in a large cohort of FRDA patients, and explore the potential causes and consequences of vitamin D deficiency in this population.

This was a retrospective analysis using records from the London arm of the European Friedreich's Ataxia Consortium of Translational Studies (EFACTS) database. Eighty‐two genetically confirmed FRDA patients with at least one serum 25‐hydroxy vitamin D (25‐OHD) measurement were identified, of which 26 were excluded due to current vitamin D supplementation. Demographic and clinical data, including disease duration, SARA (Scale for the Assessment and Rating of Ataxia) scores, and presence of falls, were collected. Because this was a service evaluation project, informed consent was not required.

Basic descriptive and clinical data can be found in Table 1. Fifty‐six patients were enrolled in the study (23 males). The median 25‐OHD level was 31.8 nmol/L (23.1–49.9, interquartile range) and did not differ significantly between male and female patients. Falls were experienced by 81.1% of patients and in terms of mobility, 55.4% of patients were wheelchair‐bound, 17.9% were ambulatory but used a wheelchair regularly, and 26.8% of patients were fully ambulatory. There were no associations between 25‐OHD levels and SARA score, disease duration, age at onset, age, falls, the presence of lower limb proximal muscle weakness or its severity, or ambulatory status.

**TABLE 1:** Subject characteristics

	Female (*n* = 33)	Male (*n* = 23)	Total (*n* = 56)	*P* value
25‐OHD level (nmol/L)	34.7 (26.5–51.9)	25.0 (16.0–46.0)	31.8 (23.1–49.9)	0.150
Disease duration (years)	17.0 (13.5–25.0)	18.0 (12.0–28.0)	17.0 (13.0–25.0)	0.900
Age (years)	31.0 (24.5–44.0)	24.0 (21.0–36.0)	30.0 (22.3–40.5)	0.070
Onset (years)	14.0 (7.0–17.0)	9.0 (4.0–14.0)	11.0 (6.0–16.8)	0.097
SARA total Score	22.0 (17.3–29.0)	25.0 (13.0–29.0)	22.0 (16.3–29.0)	0.854
Falls (*n*) *n* = 54, 31 females^1^	25 (80.6%)	18 (78,3%)	43 (81.1%)	1.0^a^
Wheelchair bound	18 (54.5%)	13 (56.5%)	31 (55.4%)	0.884

*Note*: The values of 25‐OH Vitamin D level, disease duration, age, onset and SARA total are reported as median (interquartile range). Falls and wheelchair‐bound are reported as frequency (% of total group).

^a^ Fisher's exact test used.

^1^ Data regarding falls was missing for two females.

In this study we confirmed that vitamin D deficiency is prevalent in FRDA patients.

In line with the UK National Institute for Health and Care Excellence definition we used thresholds of <50 nmol/L and <25 nmol/L to define insufficiency and deficiency, respectively. In our cohort, 48% of patients had insufficient levels of 25‐OHD, and 29% were deficient. Of note, the average 25‐OHD level within the male group was 25 nmol/L, at the deficiency threshold. According to the National Diet and Nutrition Survey, median vitamin D levels in adults ages 19–64 were 49.7 nmol/L in women and 44 nmol/L in men.^2^ Using this survey data, and matching for our cohorts gender distribution, we can hypothesize that FRDA patients are at higher risk of vitamin D deficiency than the general adult population (median 31.8 nmol vs 41 nmol). If we compare these data with those of UK individuals living with multiple sclerosis, another chronic neurological disease that affects young adults, we see a similar picture of vitamin D status (median unsupplemented 25‐OHD level of 38 nmol/L).^3^

Interestingly, vitamin D deficiency was not associated with any of the clinical measures assessed including SARA score or ambulatory status. Since the assumption is that deficiency is primarily linked to limited sunlight exposure, which one would expect with higher levels of disability, this finding requires further explanation. Of note, calcitriol (the active form of vitamin D) has been shown to increase frataxin levels both in FRDA cell lines and in patients, suggesting a more nuanced relationship between the disease and vitamin D status.^4^

Given the established benefits for bone health, particularly in a cohort in which falls are highly prevalent, coupled with the low cost and good safety profile of supplementation, we recommend that vitamin D deficiency should be screened for and actively managed in all FRDA patients.^5^

## Author Roles

(1) Research project: A. Conception, B. Organization, C. Execution; (2) Statistical Analysis: A. Design, B. Execution, C. Review and Critique; (3) Manuscript Preparation: A. Writing of the first draft, B. Review and Critique.

Z.F.: 1A, 1B, 2C, 3A, 3B.

G.T.B.: 1B, 1C, 2C, 3B.

H.G.M.: 2A, 2B, 2C, 3B.

A.C.: 2B, 2C, 3B.

P.G.: 1A, 1B, 2C, 3B.

## Disclosures

**Ethical Compliance Statement:** The authors confirm that the approval of an institutional review board was not required for this work, and informed patient consent was not necessary. We confirm that we have read the Journal's position on issues involved in ethical publication and affirm that this work is consistent with those guidelines.

**Funding Sources and Conflict of Interest:** UK MRC, Reata Pharmaceutical, NIHR. All authors have no conflict of interest.

**Financial Disclosures for the previous 12 months:** AC received no financial support in the last 12 months; PG received funding from Reata Pharmaceuticals, Cure DRPLA and the UK Medical Research Council and NIHR in support of ZF, HGM, GTB respectively.

## Data Availability

The data that support the findings of this study are available on request from the corresponding author. The data are not publicly available due to privacy or ethical restrictions.
